# Pathologic light chain amyloidosis oligomer detection in urinary extracellular vesicles as a diagnostic tool for response and progression of disease

**DOI:** 10.3389/fonc.2022.978198

**Published:** 2022-10-04

**Authors:** Shawna A. Cooper, Christopher J. Dick, Pinaki Misra, Nelson Leung, Carrie A. Schinstock, Marina Ramirez-Alvarado

**Affiliations:** ^1^ Department of Biochemistry and Molecular Biology, Mayo Clinic, Rochester, MN, United States; ^2^ Division of Nephrology and Hypertension, Mayo Clinic, Rochester, MN, United States; ^3^ Division of Hematology, Mayo Clinic, Rochester, MN, United States; ^4^ Department of Immunology, Mayo Clinic, Rochester, MN, United States

**Keywords:** amyloid, light chain (AL) amyloidosis, urine, urinary extracellular vesicles, oligomer, immunoglobulin light chain, cross reactivity, diagnostic test development

## Abstract

Light Chain (AL) Amyloidosis is a plasma cell dyscrasia producing amyloidogenic light chains (LC) that misfold and form amyloid deposits that cause damage in vital organs, primarily the heart and kidneys. Urinary extracellular vesicles (uEVs) are nanoparticles produced by renal epithelial cells throughout the nephron. We previously showed that uEVs from active renal AL amyloidosis patients contain LC oligomers that are large (>250kDa), resistant to heat and chemical denaturation, but of low abundance. Renal dysfunction in AL amyloidosis results in high urine protein, compounding technical challenges to use uEVs as analytical tools. In this study, we assess the use of uEVs as analytical diagnostic tools for response and disease progression in AL amyloidosis. Our results suggest that uEV protein concentration, urine volume, and particle concentrations are not directly correlated. Multiple strategies for overcoming non-specific antibody binding in uEV samples were validated in our study. We demonstrated that the sensitivity for pre-clinical testing is improved with a urine sample requirement algorithm that we developed. The findings of our study will provide a pathway toward development of critically needed tools for patient management. Sensitive detection of LC oligomers from a non-invasive urine sample rather than an invasive renal biopsy will reduce patient burden and healthcare costs. The ability to detect LC oligomers in patients with renal progression, despite positive hematologic response; will allow clinicians to confidently treat, but not overtreat, patients at risk of ongoing significant renal injury.

## Introduction

Urinary extracellular vesicles (uEVs), including but not limited to exosomes and microvesicles, are lipid bilayer encased nanoparticles released by the epithelial cells lining the nephron of the kidney and urinary tract (1–3). Proteins incorporated into these vesicles from the originating cells can be used to determine the nephron region of origin (1, 2). Years ago, we became interested in understanding the possible role of uEVs in light chain (AL) amyloidosis. We initially showed that AL amyloidosis patients with active disease generate high molecular weight oligomers of monoclonal, amyloidogenic immunoglobulin light chains, which can be detected with high sensitivity *via* immunoassay, as well as monoclonal light chain detection by mass spectrometry (MASS-FIX) in their uEVs as an active disease biomarker (4, 5). Notably, this work also featured several samples from a patient with unexplained renal progression despite hematologic response to treatment and demonstrated that LC oligomers could be sensitively and specifically detected on uEVs *via* both methods. As MASS-FIX is a highly technical process, immunoassay-based methods provide an attractive alternative for moving these findings toward development of a sorely needed additional tool for monitoring disease activity and renal response in AL amyloidosis.

The use of uEVs as a source of clinically relevant biomarkers has several advantages, but also critical technical considerations. Renal dysfunction in AL amyloidosis patients results in proteinuria, consisting mostly of albumin and immunoglobulin molecules. These complicate the detection of much rarer LC immunoglobulin oligomers. Urine protein concentrations are also highly variable, so sample standardization is critical to prevent false negative results due to inadequate starting material.

The aims of this study were to 1) identify any possible technical barriers to the use of uEVs as a biomarker of AL amyloidosis disease activity, and 2) determine strategies to overcome these technical considerations. Critically, we found that determination of non-albumin uEV protein and development of an algorithm for urine sample volume requirements were key to reliable detection or ruling out of nanoparticle biomarkers. Additionally, high levels of protein and the presence of urinary immunoglobulins lead to significant non-specific binding in immuno-affinity assays, which can be overcome *via* pre-conjugated or conformational epitope specific secondary antibodies.

## Results

We identified 2 pressing technical concerns for moving our pre-clinical observations of uEV oligomer detection correlating with AL disease activity toward application as a clinical assay. The first is the issue of sample and assay standardization and the second is the inherent potential for immunoglobulin crossreactivity within an immunoassay. These issues were addressed in parallel and results are presented below.

### A) Urine sample and uEV assay parameters

**Figure 1:**
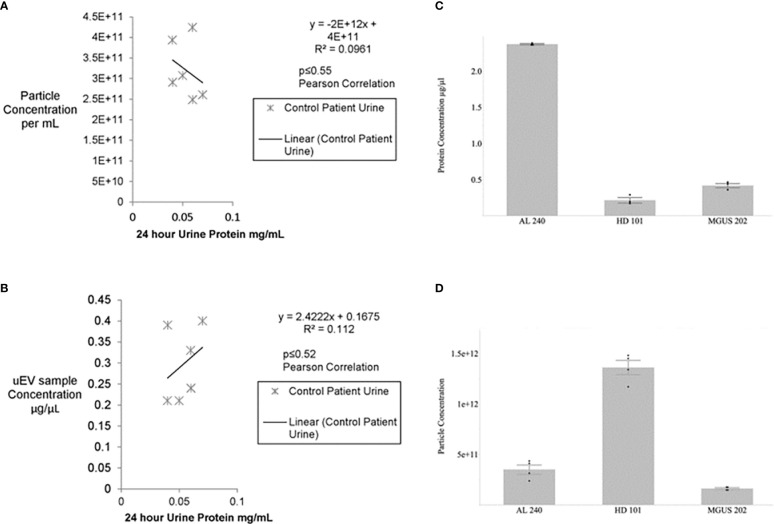
No significant association between urine protein concentration and particle number or uEV protein content for control patients **(A, B)**. Urines from healthy potential kidney donors were clinically assayed for 24 hour protein and the uEV preparation for total protein and particle numbers *via* Bradford and NTA, respectively. No significant correlation is seen between particle number and 24 hour protein or between isolated uEV sample protein and 24 hour urine protein for healthy donors. Non-parametric Spearman’s rank correlation gives p≤0.27 and p≤0.30, respectively. Similarly, a Pearson correlation for the calculated R2 values yields p<0.55 and p<0.52. Protein concentration and particle concentration are inversely correlated in plasma cell dyscrasias **(C, D)**. Newly diagnosed Light Chain Amyloidosis patient (AL 240), Monoclonal Gammopathy of Undetermined Significance (MGUS 202), and Healthy Donor (HD 101) had urinary extracellular vesicle samples assayed for total protein by Bradford Assay and particle concentration determined by Nanosight Tracking Analysis (NTA). Values are based on 3 technical replicates per patient sample and data are mean±standard error.

**Figure 2:**
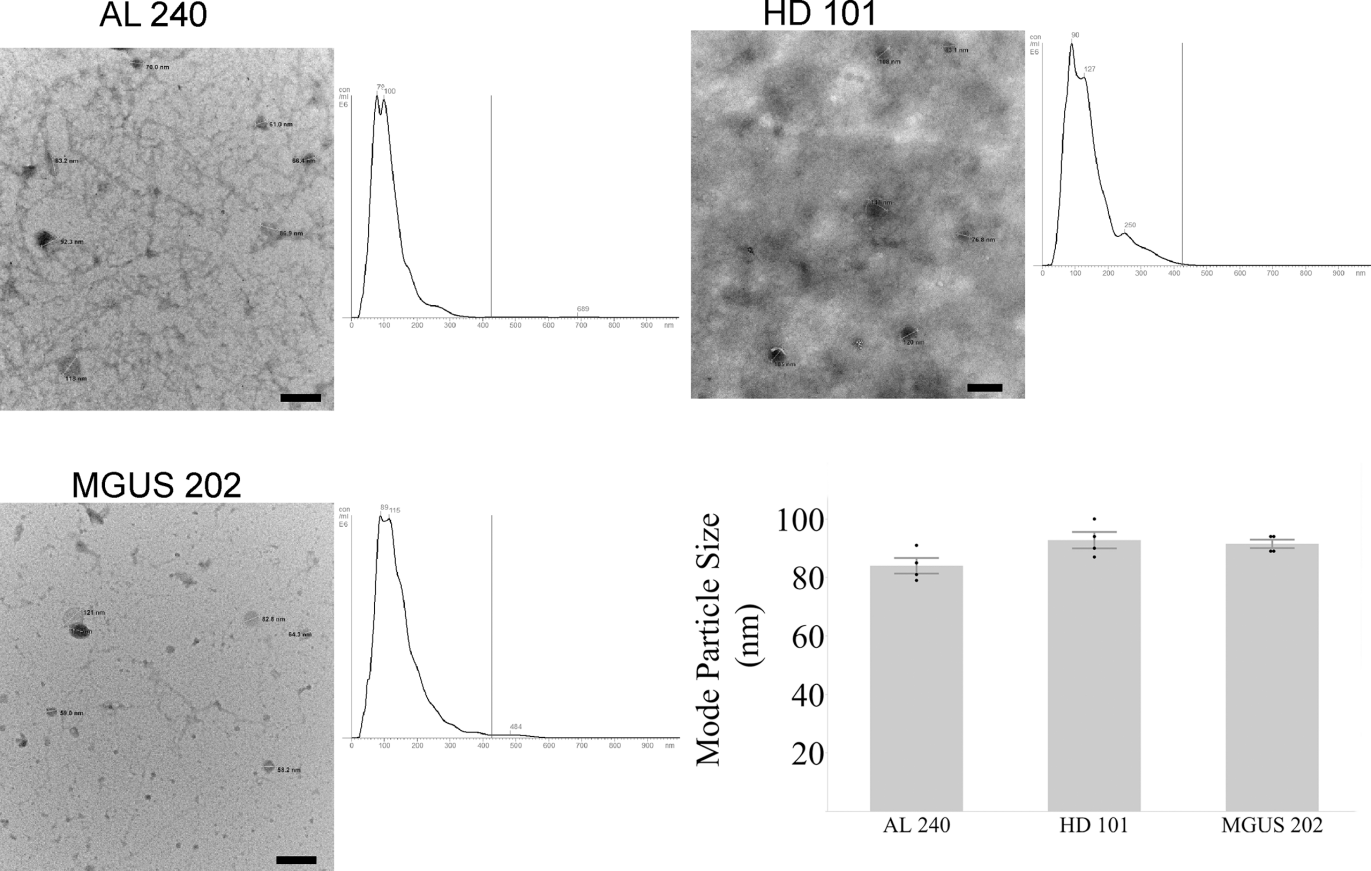
Agreement between NTA and Electron Microscopy for particle size for newly diagnosed Light Chain Amyloidosis patient (AL 240), Monoclonal Gammopathy of Undetermined Significance (MGUS 202), and Healthy Donor (HD 101). Particle sizes for the three characterized samples fall within the expected diameter size for exosomes. Scale bar is 200 nm.

The first hurdle to move a uEV-based immunoassay out of preclinical testing is sample standardization. Highly variable urine concentrations, available volumes, and patient characteristics made determination of a method to standardize the urinary extracellular vesicles recovered and used for testing a priority. We began tabulating clinical and biochemical data as shown in **Table 1**. Then we measured a number of standard parameters used to characterize extracellular vesicles within the field (6–10). Based on the data shown in **Table 1** and **Figures 1A, B** show that protein amounts in the urine and uEV prep of healthy controls are not directly correlated with the uEV particle concentration in a reliable way for sample standardization. **Figures 1C, D**, **2** show the comparative analysis of samples from an unaffected control (healthy donor 101, HD-101), a monoclonal gammopathy of undetermined significance patient (MGUS) MGUS-202, and active AL amyloidosis patient AL-240. **Figures 1C, D** show that protein concentrations do not correlate with the number of particles in the preparation. Our unaffected control HD-101 presents the lowest protein concentration and the largest amount of uEV particles. By contrast, active AL amyloidosis patient AL-240 presents the largest protein concentration and the lowest number of uEV particles. We were not surprised by the large protein concentration in uEVs from AL-240 but we were surprised to see the comparatively low numbers of uEV particles. This led us to realize that particle count was not going to be a viable parameter for sample standardization. Using transmission electron microscopy and NTA, we confirmed that the uEV size distribution (measured by the diameter of the particles) is consistent (**Figure 2**). *Thus, there were no immediately obvious metrics that would serve to standardize the samples.*

**Table 1:** Clinical characteristics of patients studied.

Patient ID	Total urine volume	Urine protein mg/day	Urine % Albumin	uEV protein Bradford μg/uL	uEV particles per mL by NTA	Heme response	FLC mg/dL / FLC Ratio	Kidney response	Scr mg/dL	uEVoligomers>250kDa
NLKD1	3095	124	–	0.39	2.905E+11	n/a	n/a	normal	–	n/a
NLKD2	2963	178	–	0.24	2.483E+11	n/a	n/a	normal	–	n/a
NLKD3	1932	77	–	0.21	3.93E+11	n/a	n/a	normal	–	n/a
NLKD4	2838	170	–	0.33	4.24E+11	n/a	n/a	normal	–	n/a
TP140	2120	106	–	0.21	3.07E+11	n/a	n/a	normal	–	n/a
TP148	3037	213		0.4	2.60E+11	n/a	n/a	normal	–	n/a
HD101-B HD101	spot spot	- -	- -	0.66 0.22	2.02E+12 1.36E+12	n/a	n/a	normal	- -	NO NO
MGUS 202	1078	1092	–	0.42	1.62E+11	Newly Diagnosed	39.2 / 0.04	Dialysis	5.9	NO
AL240	1974	8350	–	2.38	3.50E+11	Newly diagnosed	86.7 / 2.4x10-4	Newly diagnosed	1.2	YES
AL263	1179	212	13	1.42	–	Newly diagnosed	93.4 / 111	Newly diagnosed	0.9	YES
AL250	1008	2087	83	2.17	1.53E+12	Newly diagnosed	39.2 / 0.04	Newly diagnosed	0.8	NO*
ALD64E	2340	15561	57	4.24	–	Ongoing treatment/ Complete Response	9.15 / 1.06	Worsened	1.9	YES
ALD64F	2039	15843	54	3.26	5.69E+11	Ongoing treatment/ Complete Response	4.57 / 1.15	Worsened	2.1	YES
ALD64G	1728	15431	55	4.52	1.01E+12	Complete Response	6.56 / 1.43	Worsened	2.5	YES

Normal Live Kidney Donor (NLKD), Kidney Transplant patient (TP), Light chain (AL) amyloidosis, Monoclonal gammopathy of undetermined significance (MGUS), Healthy donor (HD), Not Applicable (n/a), Not available (-), Free light chain (FLC), Serum creatinine (Scr). Heme and kidney response were evaluated as described previously (5). *Inadequate sample available to meet minimum threshold for detection.

Serial samples from ALD64 highlight the presence of LC oligomers correlating with disease activity indicated by the climbing creatinine despite hematologic Complete Response and relatively stable total urine protein.

### B) Algorithm development

Due to the high variability of patient urine volumes, proteinuria, proportions of protein species present, uEV particle and protein concentrations, as well as, the inability to directly correlate hematologic response to disease activity for all of our AL amyloidosis patients, we needed to develop a novel method of reliable sample standardization. To do this, we conducted 2 years of longitudinal sampling and analysis on one renal AL amyloidosis patient with consistent renal progression and compared that data with information from additional AL amyloidosis patient samples.

We found that our method of uEV isolation does result in uEV preparations with protein concentrations that are approximately 0.3% of the 24-hour urine protein concentration for our patient population (**Table 2**). While there are patient samples in which we recover a higher percentage of total urinary protein in the uEV sample, this value provides a conservative estimate that can be used *a priori* to calculate the amount of urine necessary for reliable use of the uEV assay. This is relevant because, in our experience as a tertiary care center, many patients mail in urine samples or have other reasons for limited urine available for testing. Thus, it is critical to determine sample requirements to ensure valid results and appropriately prioritize sample and resources. This may apply to other clinical practices as well.

**Table 2:** Serial samples from a single AL amyloidosis patient demonstrating consistency of uEV protein recovery and algorithm values to determine the amount of urine to process and sample necessary for detection of AL light chain oligomers.

ID	ALD64E	ALD64F	ALD64G	ALD64H
**Total Urine Volume (mL)**	2,340	2,039	1,728	4,307
**Protein mg/day**	15,561	15,843	15,431	14,428
**% Albumin**	57	54	55	53
**Urine Total Protein Concentration (mg/mL)**	6.65	7.77	8.93	3.35
**Urine Sample (mL)**	60	60	60	60
**Total Protein in Urine Sample (μg)**	399,000	466,200	535,800	201,000
**uEV Protein Bradford (μg/μL)**	4.24	3.26	4.52	1.57
**Resuspension Volume (μL)**	375	375	375	375
**Total uEV sample Protein (μg)**	1,590	1,222	1695	589
**Ratio uEV sample to Total Urine Protein (%)**	0.3985	0.2622	0.3163	0.2929
**Non-Albumin uEV protein (μg/μL)**	1.8232	1.4996	2.0340	0.7379
**uEV Sample Volume (μL) Needed for 15μg Assay**	8.23	10.00	7.37	20.33
**Urine Volume(mL) Needed for 15μg Assay**	65.51	52.41	46.62	118.98

Approximately 0.3% of the total urine protein is recovered in the uEV ultracentrifugation sample preparation. The assay is optimally run at a concentration of 15µg of non-albumin uEV protein per 10µL of sample. By determining the 0.3% recovery rate and *a priori* knowledge of a patient’s 24-hour urine protein concentration and albumin percentage, an algorithm was developed to allow for the calculation of the amount of urine necessary to process to recover sufficient protein for study. The 0.562µg of non-albumin protein in **Equation 1**, is based on a uEV resuspension volume of 375 µL. Sample ALD64H contained an equivalent amount of protein as prior samples, but approximately double the urine volume. The algorithm determined that the standard 60mL was not sufficient and more urine was needed to run the assay appropriately.

**Figure 3:**
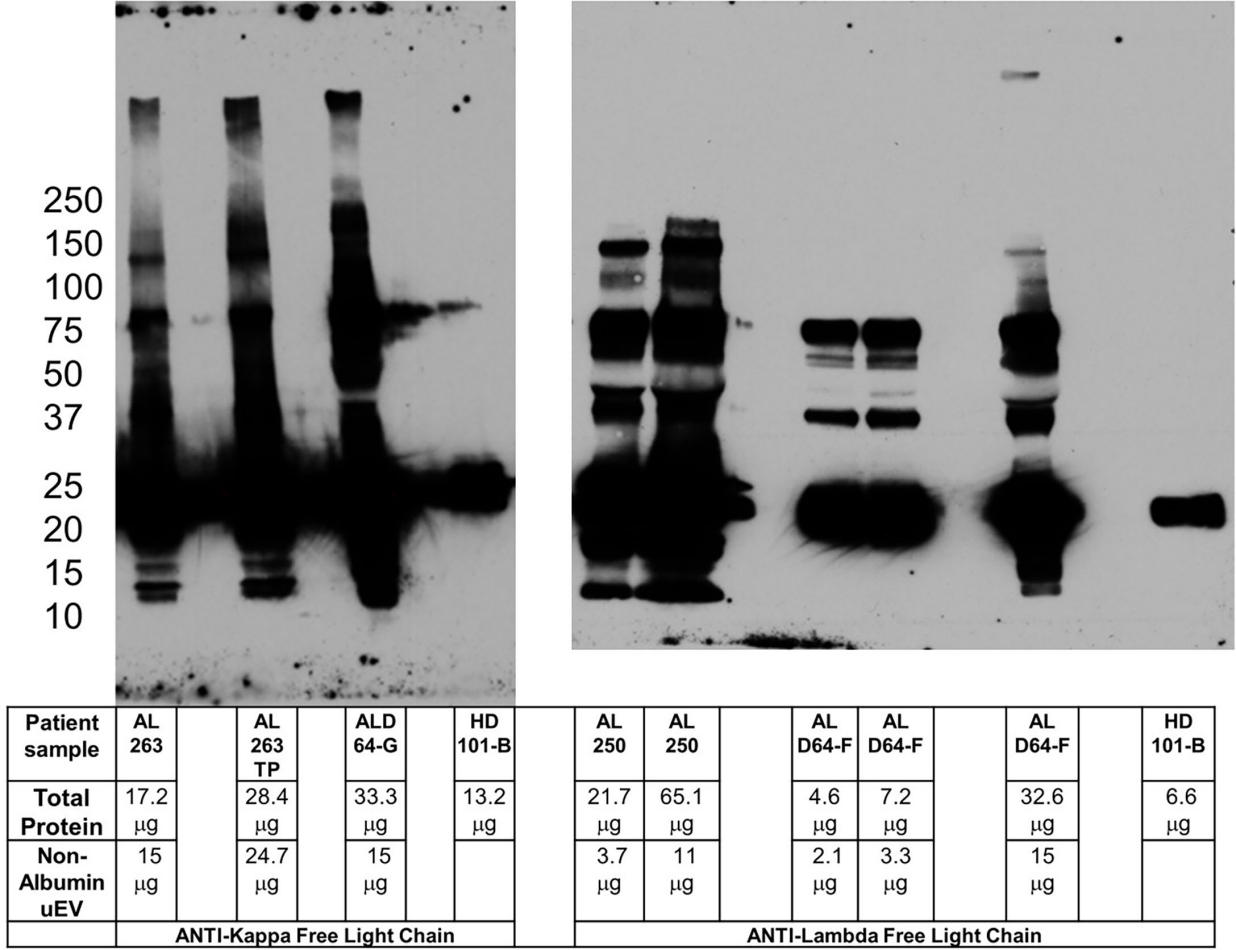
Pre-incubation of primary and secondary antibodies allows for amyloid oligomer detection. Blots for kappa (left panel) and lambda (right panel) free light chain oligomers utilizing pre-incubation of primary and secondary antibody to reduce potential non-specific binding. The clinical characteristics of the patients sampled are listed in **Table 1**. Kappa sample AL 263TP is normalized to total protein in ALD64-G. Comparing lambda sample AL 250 with ALD64-G demonstrates that total protein is less important than non-Albumin uEV protein for detection of oligomers. 15μg of non-uEV protein is required to detect oligomers in active disease. Healthy control samples are loaded as maximum amount the well will hold because the urine inherently contains less protein.

After extensive trials documented in ***section C*** and **Figure 3**, we determined that for our LC oligomer detection assay, a minimum of 15µg of non-albumin uEV protein was the threshold amount needed to detect LC oligomers. *Thus, the amount of non-albumin uEV protein recovered in our uEV isolation preparation was the most meaningful value for standardizing samples.*

Therefore, the knowledge that approximately 0.3% of the total urinary protein is recovered in the uEV preparation, the urine protein concentration, the % albumin, and our data demonstrating a requirement for at least 15µg of non-albumin uEV protein, has allowed us to develop an algorithm to calculate the amount of processed urine to obtain an appropriate uEV sample, as well as the amount of uEV sample to run in the assay for reliable results. This has been summarized into the algorithm presented as Equation 1 with data from **Table 2**.

This assay and algorithm are unique because we have overcome the technical obstacles described above by shifting the paradigm for assay standardization to a personalized value rather than ‘one size fits all’. Broad application of this approach would actually be remarkably easy due to the fact that the input values to the algorithm are already collected as standard of care and laboratory practice, but are being utilized in a novel way.

### C) Mitigation strategies for immunoglobulin crossreactivity

It has been increasingly demonstrated that extracellular vesicle preparations from urine, serum and/or plasma can present significant immunoglobulin contamination that can complicate the analysis due to immunoassay cross-reactivity (11, 12). AL amyloidosis presents an additional challenge as the pathological protein and proposed biomarker is an immunoglobulin as well. Indeed, the presence of immunoglobulins in uEV samples can cause issues of assay interference, particularly non-specific binding of secondary antibodies during Western Blot analysis (13).

The problem of immunoglobulin interference is two-fold. First, the size of immunoglobulin heavy chain (50kDa) and immunoglobulin light chain (25kDa) overlap in size with a multitude of other proteins that may be of interest for uEV characterization. For our work, the preferable protein markers for determining the nephron region of origin for our uEVs are glomerular podocin (42kDa), tubular AQP1 (25-28kDa, glycosylated 40-45 kDa) and collecting duct AQP2 (26kDa) which overlap almost directly with immunoglobulin heavy and light chains. Non-specific binding of our secondary antibodies to immunoglobulin confounds our ability to blot for these proteins and has been reported as a problem by other groups as well (14). Second, detection of immunoglobulin light chain oligomers relies on antibody specificity and confidence that the multiple species detected on a blot are truly complexes of immunoglobulin light chain and not the result of non-specific binding due to the high protein concentrations required to detect low abundance oligomers.

The potential to utilize any protein (or nucleic acid) biomarkers from nanoparticle EVs for eventual clinical use is dependent on a high specificity in the detection method. This became apparent in our study when we switched to a new lot of secondary antibodies. We found that the use of the new secondary antibody preparation allowed us to identify the presence of 75 and 100kDa bands with HD-101, which contrasted with our previous work with unaffected, healthy controls. Western blotting with no primary, in addition to the testing of three different secondary antibodies confirmed that these antibodies were binding non-specifically to uEV immunoglobulin molecules and not providing a true assessment of oligomeric light chain content in uEVs (**Figure S1**). This included a secondary antibody specifically listed as pre-adsorbed to minimize human immunoglobulin cross-reactivity, (Rabbit anti-Sheep IgG [Abcam ab7111)]. While this antibody may be a good choice for other applications, it turned out not to be ideal for our uEV analysis of oligomeric light chains.

Solving these two issues of secondary antibody specificity required two distinct approaches. For proteins for which extreme denaturation is not an issue for detection, we utilized a conformational epitope binding secondary antibody (Veriblot™, Abcam ab131366). This secondary antibody binds only to immunoglobulin molecules in the native (folded) state (in the case of western blot, the primary antibody) and relies on western blotting conditions in which the samples are fully denatured. This secondary antibody helps to reduce cross-reactivity when blotting samples with immunoglobulins in denaturing gels, such as SDS-PAGE, and after immunoprecipitation steps.

**Figure S2** demonstrates the specificity of the Veriblot secondary antibody with samples run with or without primary antibody. Crude and fractionated samples were run in panel A without primary (Veriblot secondary antibody only) and resulted in no signal, as expected. The same amount of protein loaded for the same preparation is shown to result in appropriate signal in panel B, when blotted for IgG heavy chain and the same Veriblot secondary antibody conditions. This demonstrates that immunoglobulin is not causing non-specific binding for this healthy control patient. Similarly, in panel C and D, two of our glomerular markers are blotted for samples from a patient with a significant plasma cell dyscrasia and extremely high uEV protein levels. This demonstrates that this secondary antibody allows for detection of Podocin (used as a glomerular origin marker) in sucrose gradient fractions 5 and 6 and AQP 1 (tubular origin marker) in fractions 10 and 11 with appropriate blank regions in the alternate membrane, as previously reported (4, 5). Binding of secondary antibody to immunoglobulin molecules would result in false positive blots and misidentification of fractions.

The additional denaturing sample treatment required for use of Veriblot, while specific, is not ideal for our LC oligomer samples and required a different strategy. Pre-incubation of primary and secondary antibody to reduce non-specific binding is a technique used successfully for other types of Western blot studies (15). By pre-incubating and allowing binding of the secondary antibody to the primary, the secondary antibody is no longer available to bind other sample immunoglobulin molecules in a non-specific manner. The challenge for us was to determine the ideal ratio of primary to secondary to prevent excessive binding of expensive non-conjugated primary while not reducing the ability to visualize the protein of interest. **Figure 3** demonstrates the ability to use the pre-incubation technique to detect high molecular weight oligomers in a patient with active disease, but not in healthy controls or patients without renal involvement. The sample from patient AL 250 highlights the importance of non-albumin uEV protein rather than total protein for oligomer detection. **Figure S3** shows the healthy control with pre-incubated light chain antibodies, as well as the anti-IgG heavy chain with Veriblot from **Figure S2** to clearly demonstrate that immunoglobulin is present in control samples.

Thus, we have demonstrated two independent approaches to cover a variety of use cases for uEV samples that ameliorate the issue of immunoglobulin cross-reactivity.

### uEV assay monitoring of AL amyloidosis patient disease activity

As noted in **Table 1** and **Figure 3**, sample ALD64 represents a patient with renal progression despite meeting criteria for complete hematologic response to treatment. In fact, ALD64 was included in our previous publication (**Supplementary Figure 2** and **Table 2** from (5) with clinical and biochemical uEV information and results from a sample acquired before the samples presented in this study. Using this standardized uEV immunoassay, we detect oligomers indicative of disease activity that are more consistent with the renal progression noted by the increases in serum creatinine, rather than the complete hematologic response based on serum free light chain and free light chain ratio. **Figure 3** and **Table 1** also demonstrate that LC oligomer detection performed as expected in additional patients with active disease, but not in MGUS, healthy controls, or samples that lacked adequate non-Albumin uEV protein. This represents the third report from our group showing results from patients with similar clinical circumstances that have been compared to controls confirming the sensitivity of using uEVs to detect LC oligomers and assess disease progression and organ response (4, 5).

## Discussion

The ultimate goal of our study is a rigorous standardization of our pre-clinical uEV assay aiming at the development of a non-invasive and highly sensitive diagnostic tool to assess response and progression in renal AL amyloidosis. The contributions of our results to the field are threefold: 1) A novel strategy for standardizing uEV samples that could additionally be beneficial to other groups trying to utilize this highly variable and technically challenging source of biomarkers 2) Multiple independent methods to overcome immunoglobulin cross-reactivity in uEV immunoassays 3) Demonstration of the reliability of LC oligomers on uEVs to predict AL amyloidosis disease activity, including longitudinal sampling of a particularly complicated patient subset with unexplained renal progression despite hematologic complete response.

There is a significant unmet need to improve monitoring of disease progression and treatment response in AL amyloidosis. Even for patients who are adequately monitored with current standards of care, invasive bloodwork and renal biopsies present a significant burden to the patient and the healthcare system (16, 17). This highlights the need to develop less invasive tools.

Additionally, there are subsets of patients who present with unexplained renal progression despite positive hematologic response. For these more complicated patients, more sensitive assays are critical for appropriate care. For the third time, our group has demonstrated that our pre-clinical uEV immunoassay for detection of LC oligomers is sensitive and specific for renal AL disease activity (4, 5). This has reinforced the necessity and viability of this method as a potential clinical assay and prompted efforts to identify and mitigate potential hurdles to this end.

The complexity of diagnosis and monitoring in renal AL amyloidosis patients has driven our research group to turn to the study of urinary extracellular vesicles (uEVs) as a potential source of biomarkers. There are several factors which currently complicate the ability to monitor patients’ response during and after receiving treatment. Renal AL amyloidosis management requires following both progression of renal dysfunction and hematologic response; which do not always correlate. Hematologic response is defined by changes in serum free light chain (sFLC) levels with a Very Good Partial Response (VGPR) as a difference between the monoclonal and normal light chains of <4mg/dL, and a Complete Response (CR) as a normal sFLC kappa/lambda ratio (0.26–1.65) with undetectable monoclonal protein by serum and urine immunofixation (18, 19). Current clinical testing relies on changes to the sFLC kappa/lambda ratio (18). However, renal insufficiency affects the normal levels of serum free light chain, thus altering the normal value range of the ratio (20, 21). Additionally, the serum free light chain assay does not detect clonality, rather clonality is inferred when the ratio is abnormal (5, 19, 22–25), reviewed in (26). This means that pathogenic monoclonal light chains could be present and not detected by current testing if the concentration falls within the normal range. Sidana et al. have described a large series of AL amyloidosis patients with dFLC of < 5mg/dL at diagnosis who had better cardiac outcomes, but consistently more renal dysfunction (27). For these patients, following treatment efficacy by improvement in urine proteinuria and reductions in serum creatinine is not feasible and invasive renal biopsy testing is currently required (19, 22, 28).

Recently, additional ways to evaluate renal staging, progression and response criteria were compared using a Mayo Clinic cohort (29). All three sets of criteria (International Society of Amyloidosis consensus 2004 (30), Palladini (31), and Kastritis (32) performed well at and after 6 months post-treatment. These differences are important when choosing endpoints for clinical trials. Despite these incredible efforts, there are patients like those described in our study from 2017 (5) who will not be able to benefit from the biomarkers described in Drosou et al. (29). Specifically, this patient population has progression of renal injury despite positive or stable hematologic response. As the risks and side effects of the therapeutics used to treat AL amyloidosis are not trivial, aggressive, or proactive treatment for these patients is not ideal, which is why more sensitive and accurate testing is critical.

We determined that we had two primary challenges to address in order to move our pre-clinical observations of LC oligomer detection on uEVs from patients with active AL amyloidosis toward use as a valid clinical assay: standardization of samples from a highly variable and technically challenging source and overcoming immunoglobulin crossreactivity. We addressed these issues in parallel in our laboratory and throughout the results section of this manuscript.

The critical first steps of this process of rigorous uEV assay standardization required that we determine the most appropriate metric to use for assay standardization. While total protein content is one way to standardize other EV-based assays, protein content has proven problematic for uEV researchers mainly because of the presence of albumin (33, 34). For protein dyscrasias and particularly for AL amyloidosis, the disease state and the % albumin in the urine can vary widely, so calculating the amount of non-albumin protein was necessary for each sample in our study. Particle count was also not found to be a valid metric to standardize as there was no direct correlation between particle count and urine protein or uEV sample concentration. Thus, we had to get creative and test nonstandard approaches to standardization.

We overcame these technical barriers of high protein and urine variability by employing a personalized medicine approach and shifting the paradigm for standardization to *unique values per patient that meet the sensitivity threshold for the assay and developing an algorithm for urine volumes and uEV sample requirements*. Broad application of this approach is highly feasible because the input values for the algorithm are already collected as standard of care and laboratory practice. They are simply being used in a novel way. This approach will also facilitate clinical adoption of the technique because knowing the necessary quality control metrics for the assay is part of standard assay validation and are necessary to repeat periodically as updated uEV isolation methods or detection reagents are incorporated. *Thus, the threshold values determined in this study are less important than the approach.* Additionally, this approach not only improves reliability of the assay, but also prevents wasted resources or delays in care due to inadequate sample acquisition.

There are limitations to the current work. Our assay relies on an aliquot from a 24-hour urine collection. There are some ongoing efforts to eliminate the 24-hour urine proteinuria test as the gold standard for renal amyloidosis and some alternatives have been recently proposed (35). This may require an adjustment to sample standardization using our proposed method. However, the paradigm shift of using assay threshold to standardize rather than biophysical properties of EVs means that this new strategy can quickly be applied to new sample sources and allow for assay revalidation.

Additionally, the clinical characterization of AL amyloidosis patients in this study builds on over 10 years and multiple published reports of work using uEVs to assess response and progression in patients with AL amyloidosis. For example, our original uEV publication in 2012 demonstrated the specificity of LC oligomers to active patients with AL amyloidosis vs those with Multiple Myeloma, Monoclonal Gammopathy of Undetermined Significance, Membranous Glomerulonephritis, IgA nephropathy, or AL amyloidosis patients in remission with renal improvement (4). The presence of LC oligomers on uEVs was confirmed by electron microscopy, controls for IgG, and proteomics. Additionally, a number of the patients underwent renal biopsy and their results corresponded to the findings from the uEV assay. Similarly, in 2017 we demonstrated this specificity *via* uEV immunoassay with confirmation *via* MASS-FIX and MALDI-TOF. MiRAMM on serum and uEV LC also demonstrated that the same species present in serum were identified in uEVs. Additionally, 4 patients are presented with renal progression despite positive/complete hematologic responses, including ALD64; which is the same patient shown in **Tables 1**, **2**. Despite negative serum and urine immunofixation, this patient has demonstrated renal injury that has progressed over the 2 years that our group was longitudinally testing their serial samples for uEV LC oligomers (5).

Thus, these publications added to the current dataset represent almost 40 patients including longitudinal followup of patient ALD64 for over 2 years. The goal of this study was to refine technical aspects of the assay to move it out of our laboratory for future application in clinic for AL amyloidosis patients, and particularly for complex ones like ALD64.

In conclusion, there is a significant unmet clinical need for less invasive and more sensitive approaches to follow response and progression in renal AL amyloidosis. The ability to detect light chain oligomers, specifically associated with active disease has been demonstrated by our group on multiple occasions (4, 5). However, while our assay utilizes methods that are available in any clinical laboratory and utilizes samples that are already collected as standard of care, technical considerations have previously prevented our ability to confidently move our assay into clinical testing. Now that we have demonstrated the feasibility of sample standardization and multiple approaches to deal with technical considerations; this assay is poised for future integration into standard patient management strategies with minor changes in workflow and no additional samples required from the patient. Additionally, standardizing samples based on assay parameters rather than biophysical properties of the EVs represents a paradigm shift that could move the field forward and can be applied by other groups working on biomarker development for plasma cell dyscrasias and EVs in general.

## Materials and methods

### Urine samples

This study was conducted in adherence with the declaration of Helsinki and approved by the Mayo Clinic Institutional Review Board. Urine samples were obtained from clinical residual urine taken from 24-hour urine collections of plasma cell dyscrasia and renal transplant patients. HD 101 is a first of the morning void sample taken from a healthy laboratory volunteer. Urine was treated with 0.02% sodium azide and kept at 4°C until processing.

### Vesicle isolation

A urine sample (60-100 mL) was dialyzed against ddH_2_O overnight using 3.5kDa molecular weight cut-off SpectraPor membranes (Spectrum Labs, Rancho Dominguez CA). The next day, the sample was filtered through a Nalgene Rapid Flow filtration unit with a 0.2 µm aPES membrane (Thermo Scientific, Rockford IL), treated with 0.02% sodium azide, and Complete^®^ EDTA-free protease inhibitor (Thermo-Pierce, Rockford IL). The samples were then loaded into 29.9 mL Beckman Optiseal Tubes and spun at 100,000 × g on a 70 Ti fixed angle rotor in a Beckman Coulter Optima L100 XP Ultracentrifuge (Beckman Coulter, Indianapolis IN) for 90 minutes at 4°C. Samples were then resuspended in cell culture grade 1× PBS (Cellgro without calcium or magnesium Corning, Manassas VA) with protease inhibitor. Samples were stored at 4°C until use.

### Sucrose gradient fractionation

Samples were fractionated as in Ramirez Alvarado et al., 2012 with the following modifications: crude uEV samples were prepared as above and then 300 µL of sample was layered over the 5-30% D_2_O sucrose gradient (Sigma Chemical, St Louis MO) (4). Gradients were centrifuged for 24 hours using a TH641 swinging bucket rotor on a Sorvall Discovery Ultracentrifuge (Thermo Scientific) at 274,000 × g. 6mm fractions were then collected using the BioComp Gradient Station^®^ (Biocomp, Canada). Aliquots were stored at 4°C for immediate analysis and the remainder frozen at -80°C for further study.

### Nanosight tracking analysis (NTA)

Particle concentration and mode particle size was measured by Malvern NS300 (Malvern Instruments Ltd, Malvern, UK) with a 532 nm green laser and Scientific CMOS trigger camera. Nanosight NTA 2.3.5 was used for data processing.

Based on expected polydispersity, video length was set to 60 seconds for 10-40 particles per frame. The camera was set to 12, shutter set to 600, gain to 350, and detection threshold to 3. Sample viscosity was set to water and temperature to 23.3°C. The default settings were used for minimum expected particle size, minimum track length, and blur. Samples for comparison were run within the same session to minimize variation.

Samples were diluted in cell culture grade 1× PBS to obtain approximately 30 particles per frame. Samples were loaded into the sample chamber using a 1 mL Monoject tuberculin syringe (Covidien, Mansfield MA) attached to a syringe pump (Harvard Apparatus, Holliston MA). Flow rate for advancing the sample and agitating between replicates was 255 µL per minute and readings were taken at a flow rate of 25 µL per minute. Four replicate 60 second sessions were taken per syringe for each patient. Data are expressed as mean ± standard error of the mean.

### Protein determination

For **Figure 1**, protein content was quantified *via* Bradford assay using the BioRad Protein Assay Dye Reagent (#500-0006 BioRad Laboratories, Hercules CA). Briefly, the reagent was diluted 1:5 in ddH_2_O immediately prior to use and a standard curve was generated using a commercially prepared albumin standard (Thermo Scientific) for 1-5 µg/mL. 2 µL of crude extracellular vesicle prep was added to 1mL of dilute reagent and read using a SpectraMax 384 Plus Spectrophotometer (Molecular Devices, Sunnyvale CA) at 595 nm. Samples were measured in triplicate and data expressed as in µg/µL as mean ± standard error of the mean.

For samples in **Table 1**, a microplate version of the Bradford assay was used. Microplates (Greiner Bio-One Flat bottom, Frickenhausen Germany) were loaded with 200µL of the same reagent was used as above and sample volume was 10 µL. The standard curve was generated by diluting the 1mg/mL BSA standard reagent 1:5 and then making 1:10 dilute working standards to load 10µL of each for 1-5µg/mL. Crude uEV preps were diluted 1:25 in ddH_2_O to fall along the standard curve. Plates were incubated with gentle agitation for 5 minutes and then read using the SpectraMax 384 Plus Spectrophotometer at 595nm using SoftMax Pro 5.4.1 software and the Bradford protocol.

### Transmission electron microscopy

8 µL of crude uEVs were spotted onto copper grids (FCF300-CU Formvar/Carbon 300, Electron Microscopy Sciences, Hatfield PA) and allowed to dry for 90 seconds. Excess liquid was blotted off with filter paper before staining with 2% uranyl acetate (# 22400 Electron Microscopy Sciences) in sterile ddH_2_O for 45 seconds. Blotted grids were then washed with 8µL of sterile ddH_2_0 and dried for at least 5 minutes before imaging on a Phillips Tecnai T12 transmission electron microscope (FEI, Hillsboro, OR) at 80kV.

### Western blot

Proteins from crude uEV samples and sucrose gradient fractions were separated *via* SDS-PAGE. Briefly, uEV sample was heated for 5 minutes (15 minutes for Veriblot samples) at 95°C in 2× SDS loading buffer containing 4% SDS and 10% beta-mercaptoethanol. Samples were run on either 4-15% or 12% Pre-Cast Criterion Tris-HCl gels (BioRad Laboratories) and transferred to PVDF membranes (Immobilon-P, EMD Millipore, Burlington MA). Transfer was conducted on ice for 1 hour at 100V with fresh transfer buffer containing methanol. Membranes were blocked overnight at 4°C in 4% BSA with 0.01% sodium azide. Membranes were then removed from blocking buffer and washed in tris buffered saline-tween 20 (TBS-T) wash buffer 2× 5 minutes before blotting.

For **Figure S1** no primary antibody was used. All secondary antibodies were diluted in TBS-T and incubated for 1 hour at room temperature. In A) Human IgG pre-adsorbed rabbit anti-sheep HRP secondary antibody (Abcam ab7111, Cambridge MA) was diluted 1:8000. In B) goat anti-rabbit HRP secondary (Thermo 31460) was diluted 1:100,000. In C) rabbit anti-sheep HRP secondary (Thermo 31480) was diluted 1:200,000.

For **Figures S2A, B**, samples were blotted with either only Veriblot for IP secondary antibody (Abcam ab131366) diluted 1:2000 in TBS-T and incubated for 2 hours at room temperature or rabbit anti-human IgG heavy chain (Proteintech 16402-1-AP, Rosemont IL) diluted 1:2000 in 2% BSA + 0.05% sodium azide buffer for 2 hours at room temperature followed by Veriblot. For C and D, primary antibodies rabbit anti-podocin (Sigma P0372) and rabbit anti-AQP-1 (Proteintech 20333-1-AP) were diluted 1:1000 in 2% BSA + 0.05% sodium azide buffer and incubated for 2 hours at room temperature followed by Veriblot.

**Figure 3** utilized the pre-incubation of primary and secondary antibody to minimize non-specific binding. Sheep anti-human lambda free light chain (Binding Site PX018, San Diego CA) diluted 1:1000 was combined with rabbit anti-sheep HRP (Abcam ab7111) diluted 1:20,000 in TBS-T and co-incubated in a falcon tube for 2 hours at room temperature. Pre-incubation was used identically as for lambda with sheep anti-human kappa free light chain (Binding Site PX016) diluted 1:20,000 in TBS-T and rabbit anti-sheep HRP (Abcam ab7111) diluted 1:20,000. The antibodies were then added to the blot for an additional 2 hours at room temperature.

**Figure S3** utilized the blot from **Figure S2B** for the IgG heavy chain sample and the protocol from **Figure 3** for the kappa and lambda light chain pre-incubations.

Blots for all figures were visualized as follows: Membranes were washed with fresh TBS-T for 5, 15, and 20 minutes between primary and secondary antibodies and also prior to developing with SuperSignal West Pico ECL reagent (Thermo) according to manufacturer instructions. Images were captured on film (Classic Blue Autoradiography, MidSci St. Louis) and developed with on a RP X-omat system (Eastman Kodak, Rochester NY).

### Statistical Analysis

Statistical analyses were conducted using Graph Pad Prism (Graph Pad Software, La Jolla CA). Nonparametric analysis was conducted when samples failed to meet assumptions for normality. Significance level was set at p<0.05.

### Equations


$$ml\;urine\;required=\frac{0.562mg\;nonAlb\;EV\;protein}{\left(0.003\;\times \%\frac{nonAlb}{100}\right)\left(total\;urine\;protein\;concentration\frac{mg}{mL}\right)}$$

## Data availability statement

The original contributions presented in the study are included in the article/**Supplementary Material**. Further inquiries can be directed to the corresponding author.

## Ethics statement

The studies involving human participants were reviewed and approved by Institutional Review Board. Written informed consent for participation was not required for this study in accordance with the national legislation and the institutional requirements.

## Author contributions

SC, NL, CS, and MR-A designed the study. SC, CD, and PM conducted the experiments. SC, CS, NL, and MR-A analyzed the data. SC and MR-A wrote the initial draft of the manuscript. All authors contributed with editions and approved the final version of the manuscript.

## Funding

This work was supported by the Mayo Clinic Department of Laboratory Medicine and Pathology Small Grants (MR-A, NL), the generous support from Dr. Fernando Cosio (MR-A), the Obaid fund (CS), the Mayo Foundation, and the generous support of amyloidosis patients and their families.

## Acknowledgments

We wish to thank the patients of the Plasma Cell Dyscrasia clinic who volunteered their samples for research. The Mayo Clinic Renal laboratory was invaluable for sample acquisition. Former members of the Ramirez-Alvarado laboratory, especially Dr. Luis Blancas-Mejia contributed technical training and experimental discussions. The Mayo Microscopy and Cell Analysis Core was utilized for some of the experiments performed in this report. The authors also wish to thank EB Allen for assistance with manuscript formatting and preparation.

## Conflict of interest

The authors declare that the research was conducted in the absence of any commercial or financial relationships that could be construed as a potential conflict of interest.

## Publisher’s note

All claims expressed in this article are solely those of the authors and do not necessarily represent those of their affiliated organizations, or those of the publisher, the editors and the reviewers. Any product that may be evaluated in this article, or claim that may be made by its manufacturer, is not guaranteed or endorsed by the publisher.

## References

[1] Pisitkun T Shen RF Knepper MA. Identification and proteomic profiling of exosomes in human urine. Proc Natl Acad Sci U S A (2004) 101(36):13368–73. doi: 10.1073/pnas.0403453101 PMC516573 15326289

[2] Gonzales PA Pisitkun T Hoffert JD Tchapyjnikov D Star RA Kleta R. Large-Scale proteomics and phosphoproteomics of urinary exosomes. J Am Soc Nephrol (2009) 20(2):363–79. doi: 10.1681/ASN.2008040406 PMC2637050 19056867

[3] Chen CL Lai YF Tang P Chien KY Yu JS Tsai CH. Comparative and targeted proteomic analyses of urinary microparticles from bladder cancer and hernia patients. J Proteome Res (2012) 11(12):5611–29. doi: 10.1021/pr3008732 23082778

[4] Ramirez-Alvarado M Ward CJ Huang BQ Gong X Hogan MC Madden BJ. Differences in immunoglobulin light chain species found in urinary exosomes in light chain amyloidosis (Al). PloS One (2012) 7(6):e38061. doi: 10.1371/journal.pone.0038061 22723846 PMC3377641

[5] Ramirez-Alvarado M Barnidge DR Murray DL Dispenzieri A Marin-Argany M Dick CJ. Assessment of renal response with urinary exosomes in patients with AL amyloidosis: A proof of concept. Am J Hematol (2017) 92(6):536–41. doi: 10.1002/ajh.24717 28295502

[6] Witwer KW Goberdhan DC O’Driscoll L Thery C Welsh JA Blenkiron C. Updating MISEV: Evolving the minimal requirements for studies of extracellular vesicles. J Extracell Vesicles (2021) 10(14):e12182. doi: 10.1002/jev2.12182 34953156 PMC8710080

[7] Erdbrugger U Blijdorp CJ Bijnsdorp IV Borras FE Burger D Bussolati B. Urinary extracellular vesicles: A position paper by the urine task force of the international society for extracellular vesicles. J Extracell Vesicles (2021) 10(7):e12093. doi: 10.1002/jev2.12093 34035881 PMC8138533

[8] Ayers L Pink R Carter DRF Nieuwland R. Clinical requirements for extracellular vesicle assays. J Extracell Vesicles (2019) 8(1):1593755. doi: 10.1080/20013078.2019.1593755 30949310 PMC6442087

[9] Mateescu B Kowal EJ van Balkom BW Bartel S Bhattacharyya SN Buzas EI. Obstacles and opportunities in the functional analysis of extracellular vesicle RNA - an ISEV position paper. J Extracell Vesicles (2017) 6(1):1286095. doi: 10.1080/20013078.2017.1286095 28326170 PMC5345583

[10] Lotvall J Hill AF Hochberg F Buzas EI Di Vizio D Gardiner C. Minimal experimental requirements for definition of extracellular vesicles and their functions: A position statement from the international society for extracellular vesicles. J Extracell Vesicles (2014) 3:26913. doi: 10.3402/jev.v3.26913 25536934 PMC4275645

[11] Caradec J Kharmate G Hosseini-Beheshti E Adomat H Gleave M Guns E. Reproducibility and efficiency of serum-derived exosome extraction methods. Clin Biochem (2014) 47(13-14):1286–92. doi: 10.1016/j.clinbiochem.2014.06.011 24956264

[12] McNicholas K Li JY Michael MZ Gleadle JM. Albuminuria is not associated with elevated urinary vesicle concentration but can confound nanoparticle tracking analysis. Nephrology (Carlton). (2017) 22(11);854–863. doi: 10.1111/nep.12867 27496221

[13] Bakeberg JL Tammachote R Woollard JR Hogan MC Tuan HF Li M. Epitope-tagged Pkhd1 tracks the processing, secretion, and localization of fibrocystin. J Am Soc Nephrol (2011) 22(12):2266–77. doi: 10.1681/ASN.2010111173 PMC3250208 22021705

[14] Petry FR Nicholls SB Hebert SS Planel E. A simple method to avoid nonspecific signal when using monoclonal anti-tau antibodies in Western blotting of mouse brain proteins. Methods Mol Biol (Clifton NJ) (2017) 1523:263–72. doi: 10.1007/978-1-4939-6598-4_15 27975255

[15] Ahmed R Spikings E Zhou S Thompsett A Zhang T. Pre-hybridisation: an efficient way of suppressing endogenous biotin-binding activity inherent to biotin-streptavidin detection system. J Immunol Methods (2014) 406:143–7. doi: 10.1016/j.jim.2014.03.010 24657589

[16] Hari P Lin HM Asche CV Ren J Yong C Luptakova K. Treatment patterns and health care resource utilization among patients with relapsed/refractory systemic light chain amyloidosis. Amyloid: Int J Exp Clin Invest: Off J Int Soc Amyloid (2018) 25(1):1–7. doi: 10.1080/13506129.2017.1411796 29303358

[17] Quock TP Yan T Chang E Guthrie S Broder MS. Healthcare resource utilization and costs in amyloid light-chain amyloidosis: A real-world study using US claims data. J Comp Eff Res (2018) 7(6):549–59. doi: 10.2217/cer-2017-0100 29390860

[18] Palladini G Dispenzieri A Gertz MA Kumar S Wechalekar A Hawkins PN. New criteria for response to treatment in immunoglobulin light chain amyloidosis based on free light chain measurement and cardiac biomarkers: impact on survival outcomes. J Clin Oncol: Off J Am Soc Clin Oncol (2012) 30(36):4541–9. doi: 10.1200/JCO.2011.37.7614 23091105

[19] Leung N Glavey SV Kumar S Dispenzieri A Buadi FK Dingli D. A detailed evaluation of the current renal response criteria in AL amyloidosis: Is it time for a revision? Haematologica (2013) 98(6):988–92. doi: 10.3324/haematol.2012.079210 PMC3669457 23729727

[20] Hutchison CA Harding S Hewins P Mead GP Townsend J Bradwell AR. Quantitative assessment of serum and urinary polyclonal free light chains in patients with chronic kidney disease. Clin J Am Soc Nephrol: CJASN (2008) 3(6):1684–90. doi: 10.2215/CJN.02290508 PMC2572283 18945993

[21] Palladini G Milani P Foli A Basset M Russo F Bosoni T. The impact of renal function on the clinical performance of FLC measurement in AL amyloidosis. Clin Chem Lab Med (2016) 54(6):939–45. doi: 10.1515/cclm-2015-0985 26943606

[22] Leung N Gertz MA Zeldenrust SR Rajkumar SV Dispenzieri A Fervenza FC. Improvement of cast nephropathy with plasma exchange depends on the diagnosis and on reduction of serum free light chains. Kidney Int (2008) 73(11):1282–8. doi: 10.1038/ki.2008.108 18385667

[23] Milani P Basset M Russo F Foli A Merlini G Palladini G. Patients with light-chain amyloidosis and low free light-chain burden have distinct clinical features and outcome. Blood (2017) 130(5):625–31. doi: 10.1182/blood-2017-02-767467 28546143

[24] Milani P Valentini V Ferraro G Basset M Russo F Foli A. A patient with AL amyloidosis with negative free light chain results. Clin Chem Lab Med (2016) 54(6):1035–7. doi: 10.1515/cclm-2015-0847 26677890

[25] Kyle RA Wagoner RD Holley KE. Primary systemic amyloidosis: Resolution of the nephrotic syndrome with melphalan and prednisone. Arch Internal Med (1982) 142(8):1445–7. doi: 10.1001/archinte.1982.00340210037009 7103624

[26] Karam S Leung N. Renal involvement in systemic amyloidosis caused by monoclonal immunoglobulins. Hematol Oncol Clin North Am (2020) 34(6):1069–79. doi: 10.1016/j.hoc.2020.08.002 33099424

[27] Sidana S Tandon N Dispenzieri A Gertz MA Buadi FK Lacy MQ. Clinical presentation and outcomes in light chain amyloidosis patients with non-evaluable serum free light chains. Leukemia (2018) 32(3):729–35. doi: 10.1038/leu.2017.286 28919633

[28] Palladini G Milani P Basset M Russo F Lavatelli F Nuvolone M. Urinary albumin to creatinine ratio in diagnosis and risk stratification of renal AL amyloidosis. Amyloid: Int J Exp Clin Invest: Off J Int Soc Amyloid (2017) 24(sup1):68–9. doi: 10.1080/13506129.2017.1293644 28434326

[29] Drosou ME Vaughan LE Muchtar E Buadi FK Dingli D Dispenzieri A. Comparison of the current renal staging, progression and response criteria to predict renal survival in AL amyloidosis using a Mayo cohort. Am J Hematol (2021) 96(4):446–54. doi: 10.1002/ajh.26092 33428787

[30] Gertz MA Comenzo R Falk RH Fermand JP Hazenberg BP Hawkins PN. Definition of organ involvement and treatment response in immunoglobulin light chain amyloidosis (AL): A consensus opinion from the 10th international symposium on amyloid and amyloidosis. Am J Hematol (2005) 79(4):319–28. doi: 10.1002/ajh.20381 16044444

[31] Palladini G Hegenbart U Milani P Kimmich C Foli A Ho AD. A staging system for renal outcome and early markers of renal response to chemotherapy in AL amyloidosis. Blood (2014) 124(15):2325–32. doi: 10.1182/blood-2014-04-570010 25115890

[32] Kastritis E Gavriatopoulou M Roussou M Migkou M Fotiou D Ziogas DC. Renal outcomes in patients with AL amyloidosis: Prognostic factors, renal response and the impact of therapy. Am J Hematol (2017) 92(7):632–9. doi: 10.1002/ajh.24738 28370245

[33] Merchant ML Rood IM Deegens JKJ Klein JB. Isolation and characterization of urinary extracellular vesicles: Implications for biomarker discovery. Nat Rev Nephrol (2017) 13(12):731–49. doi: 10.1038/nrneph.2017.148 PMC5941934 29081510

[34] Rood IM Deegens JK Merchant ML Tamboer WP Wilkey DW Wetzels JF. Comparison of three methods for isolation of urinary microvesicles to identify biomarkers of nephrotic syndrome. Kidney Int (2010) 78(8):810–6. doi: 10.1038/ki.2010.262 20686450

[35] Visram A Rajkumar SV Kapoor P Dispenzieri A Lacy MQ Gertz MA. Monoclonal proteinuria predicts progression risk in asymptomatic multiple myeloma with a free light chain ratio >/=100. Leukemia (2022) 36(5):1429–31. doi: 10.1038/s41375-022-01529-w PMC9064933 35190659

