# Supplementary material for: Pathologic light chain amyloidosis oligomer detection in urinary extracellular vesicles as a diagnostic tool for response and progression of disease
Source: Front Oncol. 2022 Oct 4;12:978198. doi: 10.3389/fonc.2022.978198 (PMC9577681; doi:10.3389/fonc.2022.978198)
Supplement: Supplementary file 1 [file DataSheet_1.zip › Supplementary Figure Captions.pdf]

Figure S1. Secondary antibody binding to extracellular vesicle sample immunoglobulin. No primary antibody is utilized in this blot, so the expectation is no signal unless the secondary is binding to sample immunoglobulin. Abbreviations are as follows: AL 240 Light Chain Amyloidosis patient 240; HD 101 Healthy Donor 101.
a) Abcam rabbit anti-sheep HRP secondary antibody only. Pre-adsorbed against human IgG, per manufacturer datasheet. Dilution 1:8000. Incubated 1 hour at room temperature. Film exposure time 1 minute.
b) Thermo Sheep anti-rabbit HRP secondary antibody only. Dilution 1:100,000. Incubated 1 hour at room temperature. Film exposure time 1 hour.
c) Pierce rabbit anti-sheep HRP secondary antibody only. Dilution 1:200,000. Incubated 1 hour at room temperature. Film exposure time 1 hour.

Figure S2. Veriblot Secondary Antibody Control.
a. Healthy Donor (HD 101) crude and sucrose gradient fractionated samples were blotted with only Veriblot secondary antibody to confirm a lack of binding to sample immunoglobulin.
b. The same crude extracellular vesicle prep was also probed with anti-human IgG heavy chain primary and with identical Veriblot secondary antibody conditions to confirm presence of sample IgG and Veriblot activity.
Veriblot prevents masking of 25kDa and 50kDa proteins by sample immunoglobulin. AL amyloidosis patient crude and sucrose gradient fractions were assessed for **c.** the glomerular marker podocin (42kDa) and **d.** tubular marker AQP1 (25-28kDa and 45-48kDa glycosylated). These overlap the reported sizes for IgG antibody light (23kDa) and heavy (50-60kDa) chain. Identical samples were loaded for the two blots and identical antibody concentrations (Primary 1:1000 and Veriblot Secondary 1:2000) and incubation times (2 hours at Room Temperature) used. Crude is diluted 1:10 in each blot. Podocin film exposure is 1 minute and AQP1 is 15 minutes. Podocin is expected in fractions 5-6 and AQP1 in fractions 10-11. No overlap is observed between blots in regions where immunoglobulin is expected (boxed region). X denotes an empty lane and P is the pellet from the fractionation procedure.

Figure S3. Confirmation of IgG in healthy donor HD 101 urinary extracellular vesicle samples. Anti-IgG heavy chain followed by Veriblot secondary shows IgG heavy chain and limited intact (non-denatured) IgG. Pre-incubation method detection of kappa and lambda light chains. Total protein loaded is indicated in micrograms.
