# Supplementary figures and images for: Pathologic light chain amyloidosis oligomer detection in urinary extracellular vesicles as a diagnostic tool for response and progression of disease
Source: Front Oncol. 2022 Oct 4;12:978198. doi: 10.3389/fonc.2022.978198 (PMC9577681; doi:10.3389/fonc.2022.978198)

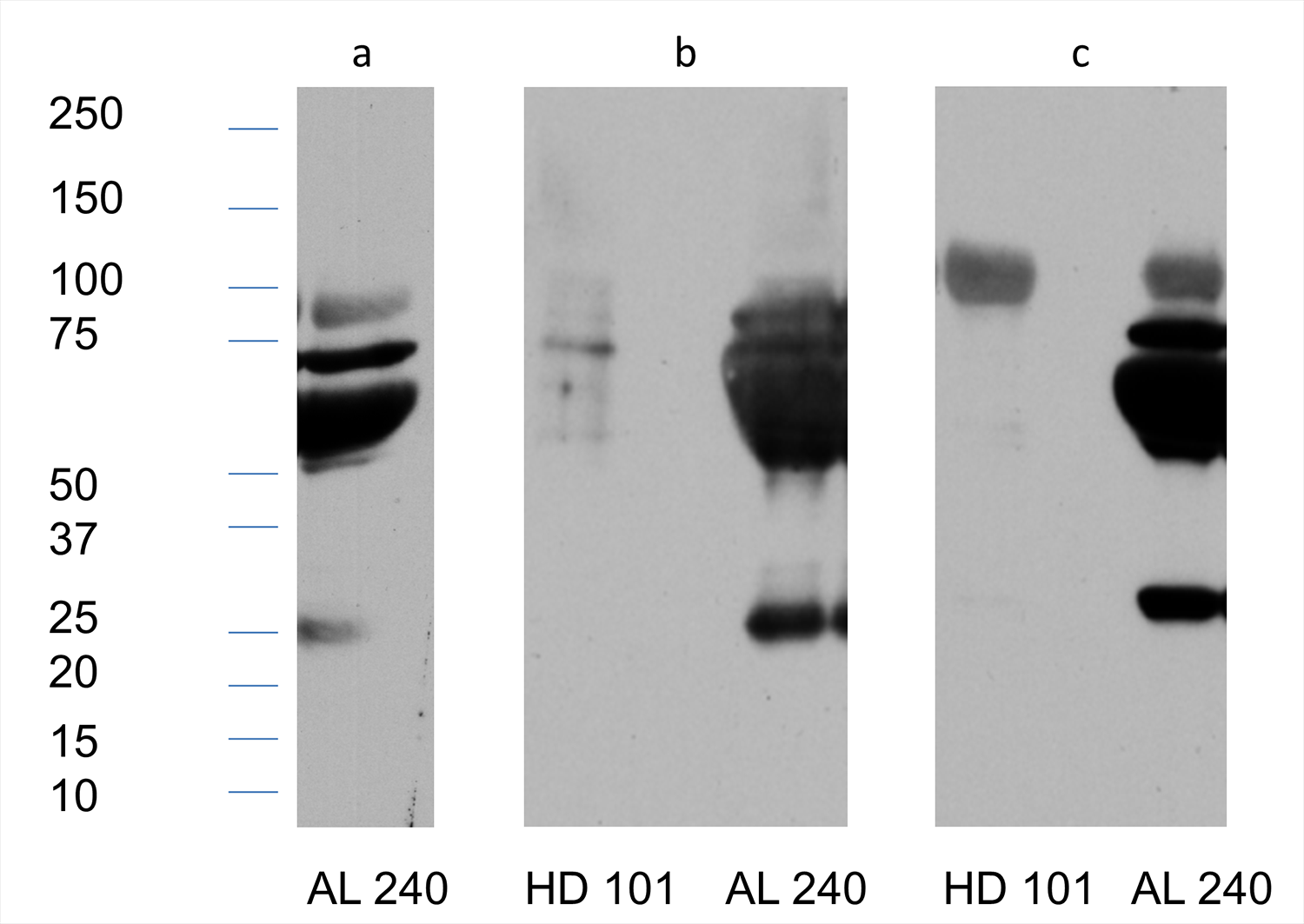

Supplement: Supplementary file 1 [file DataSheet_1.zip › Supplementary Figure S1.tif]

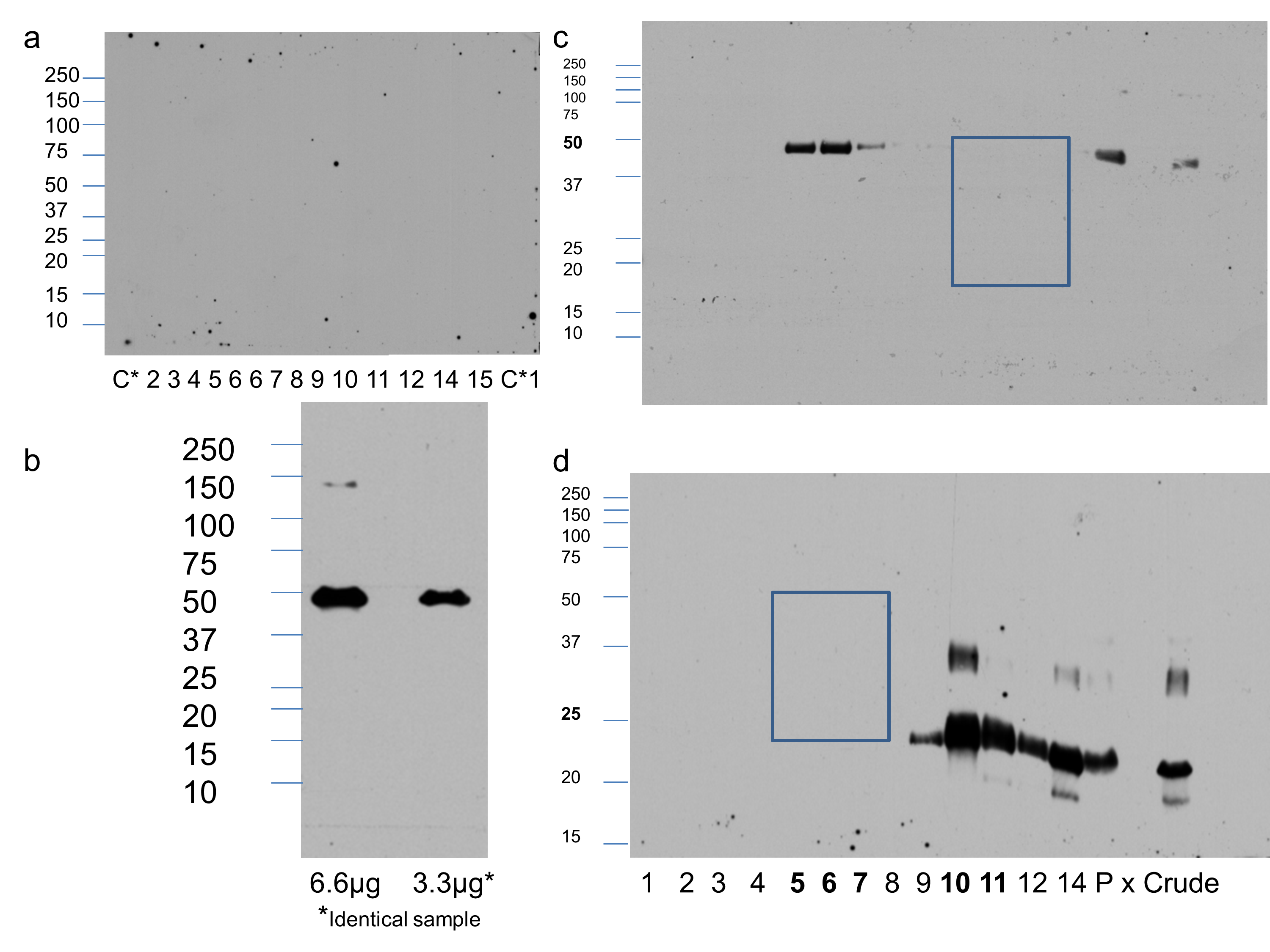

Supplement: Supplementary file 1 [file DataSheet_1.zip › Supplementary Figure S2.tif]

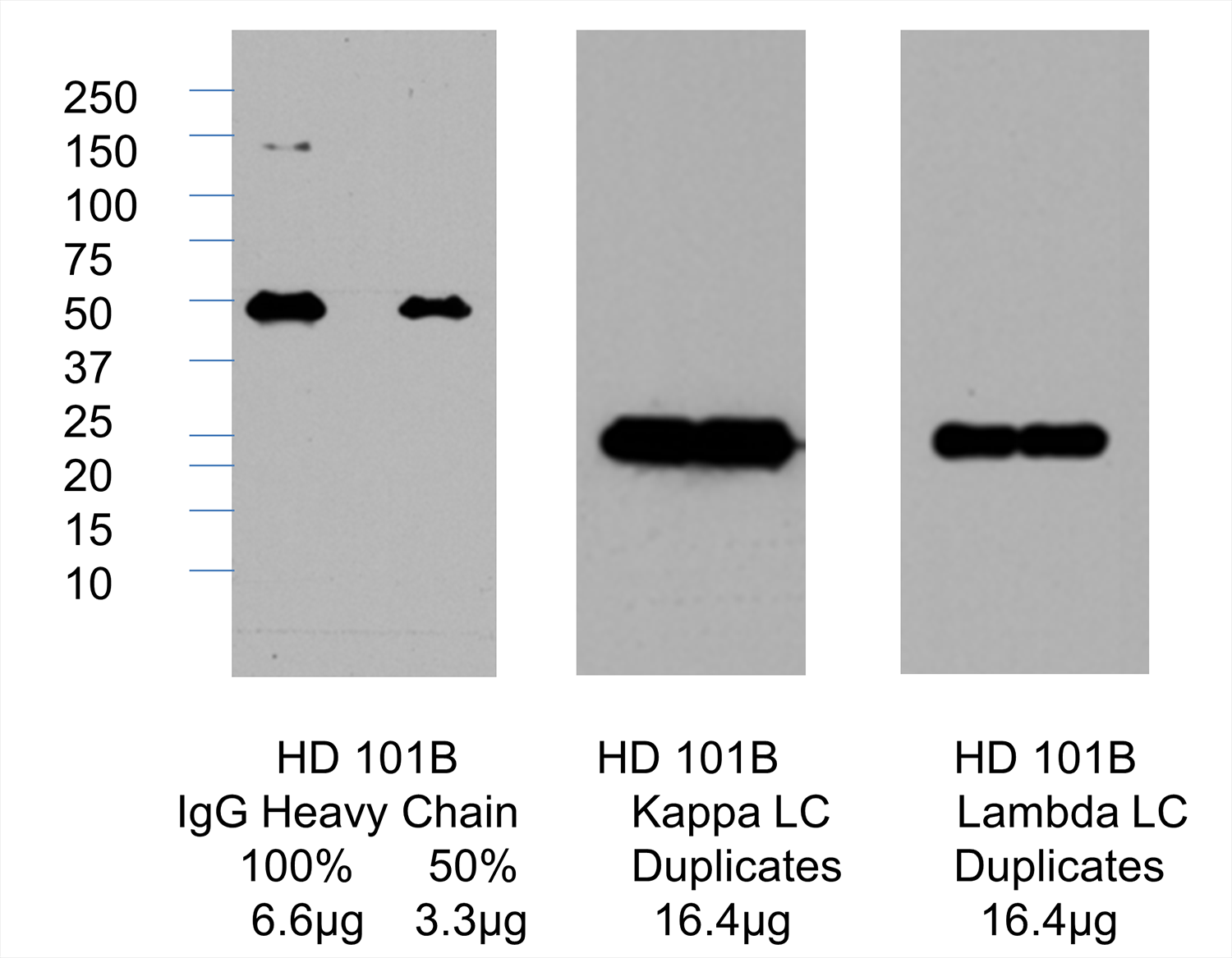

Supplement: Supplementary file 1 [file DataSheet_1.zip › Supplementary Figure S3.tif]
